# Drug Utilization Patterns and Potential Drug–Drug Interaction Burden in Respiratory Prescriptions: A Real-World Community Pharmacy Study

**DOI:** 10.3390/ph19101528

**Published:** 2026-09-26

**Authors:** Maria-Medana Drăgoi, Aimee Rodica Chis, Sebastian-Mihai Ardelean, Mihai Udrescu, Liana Suciu, Lucreția Udrescu

**Affiliations:** 1 Center for Drug Data Analysis, Cheminformatics, and the Internet of Medical Things, “Victor Babeş” University of Medicine and Pharmacy Timişoara, 300041 Timişoara, Romania; medana.tuica@umft.ro (M.-M.D.); suciu.liana@umft.ro (L.S.); udrescu.lucretia@umft.ro (L.U.); 2 Doctoral School of Pharmacy, “Victor Babeş” University of Medicine and Pharmacy Timişoara, 300041 Timişoara, Romania; 3 Department of Biochemistry, “Victor Babeş” University of Medicine and Pharmacy Timișoara, 300041 Timișoara, Romania; 4 Center for Complex Network Science, “Victor Babeş” University of Medicine and Pharmacy Timișoara, 300041 Timișoara, Romania; 5 Department of Computer and Information Technology, Politehnica University Timişoara, 300223 Timişoara, Romania; sebastian.ardelean@cs.upt.ro (S.-M.A.); mihai.udrescu@cs.upt.ro (M.U.); 6 Department II—Pharmacology, Physiology and Physiopathology, “Victor Babeş” University of Medicine and Pharmacy Timișoara, 300041 Timişoara, Romania; 7 Research Center for Experimental Pharmacology and Drug Design (X-Pharm Design), “Victor Babeş” University of Medicine and Pharmacy Timișoara, 300041 Timişoara, Romania; 8 Department I—Clinical Pharmacy and Drug Analysis, “Victor Babeş” University of Medicine and Pharmacy Timişoara, 300041 Timişoara, Romania

**Keywords:** respiratory diseases, community pharmacy, drug utilization, polypharmacy, drug–drug interactions, medication safety, real-world data

## Abstract

**Background:** Respiratory disorders are frequently managed in community pharmacies and may involve complex multidrug regimens, particularly in asthma and chronic obstructive pulmonary disease (COPD). We aimed to characterize respiratory pharmacotherapy and moderate or major potential drug–drug interaction (DDI) risk using one index respiratory prescription per patient. **Methods:** We performed a retrospective observational secondary analysis of anonymized electronic prescription data from two Romanian community pharmacies (November 2022–October 2023). From a combined source dataset comprising prescriptions with at least two active substances, we selected records containing at least one eligible respiratory diagnosis. Demographics, diagnostic groups, active substances, therapeutic classes, medication burden, and potential DDIs were evaluated. Multivariable logistic regression identified factors independently associated with at least one moderate or major potential DDI. **Results:** The final cohort included 643 patients. Median age was 52 years [interquartile range 23.5–66.0], and 50.7% were female. Asthma was the most frequent diagnostic group (41.1%), followed by acute upper respiratory tract infections (19.8%) and COPD (15.4%). The most frequent therapeutic classes were inhaled and intranasal corticosteroids (54.0%), long-acting beta-2 agonists (46.7%), and anti-infectives (35.0%). At least one moderate or major potential DDI was identified in 28.0% of index prescriptions. In the fully adjusted multivariable model, each additional active substance was associated with higher potential DDI odds (odds ratio [OR] 2.42, 95% confidence interval [CI] 1.96–3.00), while cardiovascular or metabolic co-medication showed the strongest positive class-level association (OR 14.16, 95% CI 5.25–38.21). **Conclusions:** Respiratory prescriptions showed distinct diagnostic and therapeutic profiles. The highest prevalence of moderate or major potential DDIs among the major diagnostic groups was observed in COPD. Potential DDI risk was strongly associated with medication burden and cardiovascular/metabolic co-medication, supporting targeted community pharmacy medication review that considers both respiratory therapy and the broader pharmacotherapeutic regimen.

## 1. Introduction

Respiratory diseases represent a major component of ambulatory care and community pharmacy practice. Their pharmacological management varies by diagnosis: acute respiratory tract infections may need anti-infective and symptomatic treatment, allergic rhinitis and upper-airway inflammation disorders require antihistamines or intranasal corticosteroids, while asthma and chronic obstructive pulmonary disease (COPD) often require long-term inhaled maintenance therapy with corticosteroids, long-acting beta-2 agonists, and muscarinic antagonists [1,2,3,4]. This therapeutic heterogeneity makes respiratory prescriptions a useful setting for drug utilization research, especially when diagnoses, active substances, medication burden, and medication safety outcomes are evaluated together.

Recent population data provide epidemiological context for Romania. In the 2019 European Health Interview Survey, about 2% of Romanians aged 15 or older reported asthma during the preceding 12 months [5]. For COPD, Global Burden of Disease data showed an age-standardized prevalence of about 3.3% in Romania in 2019 [6]. These estimates differ in ascertainment method and are not directly comparable with the diagnostic distribution in the current prescription-based cohort. Therefore, the relative representation of asthma and COPD in our analytical cohort should be seen as a feature of the selected respiratory prescriptions, not as reflecting the relative population prevalence of these diseases.

Community pharmacies serve as an essential link between prescribing, dispensing, patient counseling, and medication safety surveillance. In Romania, community pharmacies are privately owned pharmaceutical healthcare units open to the public, authorized by the Ministry of Health and governed by national pharmacy legislation and good pharmacy practice requirements. Community pharmacists’ statutory responsibilities extend beyond dispensing to medication information and counseling, support for rational drug use and treatment monitoring, collaboration with physicians, pharmacovigilance, and adverse drug reaction reporting [7,8,9]. Community pharmacies may contract with the national health insurance system for the provision of pharmaceutical services, including the dispensing of reimbursed prescriptions [8]. In chronic respiratory disease, pharmacist-led interventions can improve inhaler technique and adherence to inhaled therapy, identify practical problems related to medication use, and educate patients [10,11]. These aspects are clinically relevant because poor adherence and incorrect inhaler technique are frequent in obstructive respiratory diseases and may contribute to poor disease control, avoidable treatment escalation, and increased healthcare costs [10,11]. Community pharmacists play a key role in acute respiratory tract infections by managing symptoms effectively, ensuring timely referrals, and promoting antimicrobial stewardship by reducing unnecessary antibiotic use [1,12]. More broadly, pharmacy-based prescription processing can help detect medication-related problems before dispensing [13].

Drug–drug interactions (DDIs) are a significant medication-safety issue because they may diminish therapeutic efficacy, increase toxicity, and cause adverse reactions or hospitalization [14,15,16]. In Romanian community pharmacy practice, previous studies indicated that potential DDIs are frequent in routine outpatient prescribing. One study of reimbursed prescriptions from a Romanian community pharmacy reported potential DDIs in approximately one-third of prescriptions, while a statin-focused community pharmacy study found clinically relevant potential DDIs in a substantial proportion of patients treated with statins [14,15]. These findings highlight the importance of DDI surveillance at the dispensing level, particularly in patients receiving multiple drugs or cardiometabolic co-medication.

Polypharmacy is one of the strongest drivers of DDI risk. Although definitions vary, the use of five or more drugs is commonly used as an operational threshold for polypharmacy in medication-safety research [17]. In respiratory patients, polypharmacy may arise from the respiratory regimen itself, treatment of acute episodes, or comorbidities such as cardiovascular, metabolic, gastrointestinal, skeletal, or neuropsychiatric disorders [3,4]. This is especially important in COPD, where pharmacotherapy rarely occurs in isolation and is shaped by multimorbidity, older age, recurrent treatment escalation, and exposure to anti-infectives and corticosteroids during exacerbations [4,18]. Accordingly, DDI risk in COPD should be assessed at the regimen level rather than as a simple list of isolated drug pairs.

Inhaled respiratory drugs are often considered safer than systemic therapies because of their local delivery and reduced systemic exposure; however, they are not completely free from interaction risk [4]. Inhaled corticosteroids, beta-2 agonists, anticholinergic bronchodilators, and fixed respiratory combinations can lead to clinically relevant DDI risk in selected patients, particularly when combined with cardiovascular drugs, metabolic inhibitors, QT-active drugs, systemic corticosteroids, anti-infectives, or psychotropic agents [2,4]. Evidence from community pharmacy research indicates that potential DDIs involving inhaled medications may occur, especially among COPD patients with multiple comorbidities and high medication burdens [2]. Therefore, evaluating medication safety for respiratory prescriptions should include both respiratory and non-respiratory drugs.

Electronic drug interaction checkers further complicate DDI risk interpretation, as various databases and tools may identify different potential interactions and assign different severity categories to the same drug pair [19,20]. This lack of agreement creates a practical challenge for pharmacists and clinicians in identifying clinically meaningful risks among numerous alerts. Community pharmacy studies have shown that software-generated DDI alerts are frequent and may vary substantially depending on the interaction database used [19,21]. Consequently, medication safety research should not only report potential DDI prevalence, but also identify patient- and prescription-level factors associated with potential DDI occurrence that may help prioritize medication review.

Multivariable approaches are increasingly relevant in pharmacotherapy research. In asthma, multivariate analysis and machine learning methods identified exacerbation risk predictors in Romanian patients, illustrating the benefits of combining statistical modeling with data-driven techniques in respiratory research [22]. Similar approaches reported pharmacological risk stratification in other therapeutic areas, integrating polypharmacy, DDI burden, QT risk, anticholinergic burden, and other medication safety domains into clinically interpretable risk signals [23]. These studies support the potential of structured drug data to identify high-risk pharmacotherapy profiles and inform targeted medication reviews.

Despite this background, evidence integrating respiratory diagnoses, active substance prescribing, therapeutic class profiles, polypharmacy, and DDI risk in community pharmacy remains limited. Previous studies have often focused separately on inhaler technique, adherence, pharmacist intervention, antibiotic stewardship, DDI prevalence, or selected respiratory conditions [1,2,10,11,12,14,15,19,24,25,26,27]. Few studies combined diagnostic grouping, medication profiling, therapeutic pattern analysis, diagnostic–therapeutic associations, and multivariable analysis of moderate or major DDI risk within the same community pharmacy dataset. Evidence from Romanian community pharmacy practice on respiratory prescriptions, both acute and chronic, is particularly limited [3,4,25,28].

The present study addresses this gap by analyzing real-world respiratory prescriptions dispensed in two Romanian community pharmacies. Before respiratory case selection, the source dataset had been restricted to the earliest prescription by dispensing date for each patient, irrespective of diagnosis. This design provides a cross-sectional characterization of diagnostic and pharmacotherapeutic patterns represented on one retained prescription per patient, not a longitudinal assessment of individual medication exposure or total dispensing-event frequencies.

The objectives of this study are: (i) to describe the demographic and respiratory diagnostic characteristics represented in the analytical cohort; (ii) to characterize prescription-level medication use according to active substances, therapeutic classes, and treatment patterns; (iii) to evaluate associations between respiratory diagnoses and therapeutic profiles; and (iv) to identify factors independently associated with moderate or major potential DDI risk at the index prescription. By linking respiratory diagnosis, drug utilization, polypharmacy, and DDI burden within a real-world community pharmacy dataset, our study supports the role of community pharmacy data in drug safety, medication review, and risk-management strategies for patients treated for respiratory disorders.

## 2. Results

### 2.1. Derivation of the Analytical Dataset

The final analytical dataset included 643 patients, each represented by one index prescription, after respiratory case selection and complete case restriction (Figure 1). Among the 643 index prescriptions, 536 (83.36%) contained a single respiratory diagnostic code, whereas 107 (16.64%) contained multiple respiratory codes.

### 2.2. Demographic and Diagnostic Characteristics

The mean age of the analytical cohort was 46.26 years (SD 25.03), with a median of 52 years [IQR 23.5–66.0] and a range of 1–93 years. Patients aged 18–64 years constituted the largest age group (326/643, 50.70%), followed by those aged ≥65 years (178/643, 27.68%) and <18 years (139/643, 21.62%). The sex distribution was balanced, with 326 female patients (50.70%) and 317 male patients (49.30%).

Asthma was the most frequent assigned respiratory diagnostic group, accounting for 264 patients (41.06%). Acute upper respiratory tract infections were identified in 127 patients (19.75%), followed by COPD or chronic obstructive respiratory disease in 99 (15.40%), acute bronchitis or lower respiratory tract infections in 57 (8.86%), and allergic rhinitis or chronic rhinosinusitis in 43 (6.69%). Other chronic or unspecified respiratory or ENT conditions accounted for 32 patients (4.98%), influenza or pneumonia for 16 (2.49%), and other respiratory conditions for 5 (0.78%). Table 1 summarizes demographic and diagnostic characteristics.

### 2.3. Medication Burden and Therapeutic Class Distribution

The mean number of active substances recorded on the index respiratory prescription was 3.65 (SD 2.07), with a median of 3 [IQR 2–4] and a range of 2–22. The most frequent medication burden category was 3–4 active substances (263/643, 40.90%), followed by two active substances (231/643, 35.93%). 149 index prescriptions (23.17%) had five or more active substances recorded on the index prescription and therefore met the operational definition of polypharmacy (Table 2). Supplementary Table S2 presents the medication burden by assigned respiratory diagnostic group. Medication burden differed significantly across diagnostic groups (Kruskal–Wallis H = 86.79, adjusted p<0.001). COPD index prescriptions contained a median of 4 [IQR 3–6] active substances, with 39.4% containing five or more active substances, compared with a median of 2 [IQR 2–3] and 17.4%, respectively, in asthma.

The most frequently recorded active substances were formoterol (25.35%), fluticasone (21.62%), budesonide (20.06%), salmeterol (18.51%), desloratadine (13.53%), amoxicillin (13.06%), ibuprofen (12.91%), clavulanic acid (12.29%), and montelukast (9.02%). The frequencies of the most commonly recorded active substances are presented in Table 2.

Inhaled and intranasal corticosteroids (ICS/INCS) were the most frequently recorded therapeutic class, occurring in 347 index prescriptions (53.97%), followed by long-acting beta-2 agonists (LABA) in 300 (46.66%) and anti-infectives in 225 (34.99%). Antihistamine and ENT preparations were recorded in 160 prescriptions (24.88%), NSAID/analgesic/antipyretic agents in 158 (24.57%), and cardiovascular or metabolic drugs in 133 (20.68%).

Other therapeutic classes included mucolytic, antitussive, and expectorant agents in 119 prescriptions (18.51%), gastroprotective drugs in 69 (10.73%), antileukotrienes in 58 (9.02%), systemic corticosteroids in 56 (8.71%), LAMA/SAMA in 42 (6.53%), and SABA in 32 (4.98%).

### 2.4. Therapeutic Profiles by Diagnostic Group

Therapeutic class distributions varied substantially across the assigned respiratory diagnostic groups (Table 3). Asthma prescriptions were characterized predominantly by the combined ICS/INCS category (91.7%) and LABA (85.2%). In the COPD or chronic obstructive respiratory disease group, LABA (54.5%), cardiovascular or metabolic drugs (52.5%), ICS/INCS (42.4%), and LAMA/SAMA (38.4%) were frequently recorded.

Acute respiratory conditions showed a different therapeutic profile. Anti-infectives were recorded in 88.2% of acute upper respiratory tract infection prescriptions and 89.5% of acute bronchitis or lower respiratory tract infection prescriptions, together with frequent NSAID/analgesic/antipyretic and mucolytic/antitussive/expectorant use. Allergic rhinitis or chronic rhinosinusitis was characterized by frequent antihistamine and ENT preparations (88.4%) and ICS/INCS (48.8%).

Global association testing showed significant associations between the assigned respiratory diagnostic group and all evaluated therapeutic class indicators after false discovery rate adjustment. The strongest associations, based on Cramer’s *V*, were observed for anti-infectives ($\chi^{2}=427.32$, Cramer’s V=0.815), LABA ($\chi^{2}=341.46$, Cramer’s V=0.729), ICS/INCS ($\chi^{2}=296.81$, Cramer’s V=0.679), NSAID/analgesic/antipyretic agents ($\chi^{2}=210.45$, Cramer’s V=0.572), and LAMA/SAMA ($\chi^{2}=198.01$, Cramer’s V=0.555), with adjusted p<0.001 for each. These results demonstrate substantial variation in therapeutic class distribution across the assigned respiratory diagnostic groups.

### 2.5. Potential Drug–Drug Interaction Burden

We evaluated potential DDIs at the level of the index prescription after medication standardization. Among the 643 index prescriptions, 171 (26.59%) contained at least one moderate potential DDI, 45 (7.00%) contained at least one major potential DDI, and 180 (27.99%) contained at least one moderate or major potential DDI (Table 4). Because moderate and major potential DDIs could occur within the same index prescription, these severity-specific categories were not mutually exclusive.

The prevalence of at least one moderate or major potential DDI differed across the assigned respiratory diagnostic groups. The highest prevalence among the larger diagnostic groups was observed in COPD or chronic obstructive respiratory disease (52.5%), compared with 20.5% in asthma, 24.4% in acute upper respiratory tract infections, and 24.6% in acute bronchitis or lower respiratory tract infections.

Global association testing showed significant associations between the assigned respiratory diagnostic group and potential DDI burden. The association was observed for the combined moderate or major potential DDI outcome ($\chi^{2}=40.34$, Cramer’s V=0.250, adjusted p<0.001), for moderate potential DDIs ($\chi^{2}=36.13$, Cramer’s V=0.237, adjusted p<0.001), and for major potential DDIs ($\chi^{2}=27.86$, Cramer’s V=0.208, adjusted p=0.001). Thus, the prevalence of potential DDIs varied across the assigned respiratory diagnostic groups.

To characterize the composition of the major potential DDI burden, recurrent interaction pairs were examined at the index prescription level. A total of 49 major potential DDI pair occurrences were identified among the 45 patients with at least one major potential DDI. The most frequently recurring pairs included fenofibrate with rosuvastatin, clopidogrel with omeprazole, beta-blockers with theophylline, and renin–angiotensin system inhibitors with spironolactone (Table 5). Because individual major DDI pairs were infrequent, pair-level frequencies should be interpreted as recurrent prescription-level potential safety signals instead of stable prevalence estimates for specific drug combinations.

Moderate potential DDIs were more numerous and heterogeneous than major potential DDIs. A total of 422 moderate potential DDI pair occurrences were identified among the 171 patients with at least one moderate potential DDI. The most frequent pair was betamethasone with ibuprofen (22 index prescriptions, 3.42% of the full cohort), followed by indapamide with perindopril (11, 1.71%) and bisoprolol with indapamide (8, 1.24%). Other recurrent pairs involved cardiovascular drugs with inhaled bronchodilators or corticosteroids (Table 6).

### 2.6. Multivariable Analysis of Moderate or Major Potential DDI Risk

Multivariable logistic regression was used to identify factors independently associated with the presence of at least one moderate or major potential DDI at the index prescription (Table 7). Full coefficient estimates for Model A are presented in Supplementary Table S3. In Model A, which included age group, sex, assigned respiratory diagnostic group, and the number of active substances, medication burden was the strongest independent correlate of the outcome. Each additional active substance was associated with higher odds of at least one moderate or major potential DDI (OR 2.69, 95% CI 2.28–3.17, p<0.001). Compared with asthma, COPD or chronic obstructive respiratory disease was also associated with higher odds in Model A (OR 2.90, 95% CI 1.45–5.77, p=0.003).

Model B, which additionally incorporated therapeutic pattern indicators and comorbidity-related medication classes, showed improved fit relative to Model A, with lower AIC and BIC values and a higher McFadden pseudo-$R^{2}$ (Table 7). Medication burden remained strongly associated with the outcome: each additional active substance was associated with 2.42-fold higher odds of at least one moderate or major potential DDI (95% CI 1.96–3.00, p<0.001). Cardiovascular or metabolic co-medication showed the strongest positive association (OR 14.16, 95% CI 5.25–38.21, p<0.001).

Compared with asthma, higher adjusted odds of at least one moderate or major potential DDI were observed for other chronic or unspecified respiratory or ENT conditions (OR 9.80, 95% CI 2.80–34.37, p<0.001), acute upper respiratory tract infections (OR 7.60, 95% CI 2.21–26.04, p=0.001), COPD or chronic obstructive respiratory disease (OR 5.31, 95% CI 2.04–13.86, p<0.001), and acute bronchitis or lower respiratory tract infections (OR 5.26, 95% CI 1.32–20.88, p=0.018).

Age ≥65 years was inversely associated with the outcome compared with age 18–64 years (OR 0.30, 95% CI 0.13–0.68, p=0.004). The mucolytic/antitussive/expectorant pattern (OR 0.41, 95% CI 0.20–0.83, p=0.013) and LAMA/SAMA use (OR 0.29, 95% CI 0.09–0.98, p=0.046) also showed inverse adjusted associations. These findings should not be interpreted as protective pharmacological effects and may reflect the interdependence of diagnostic and therapeutic profiles within the dataset.

Variance inflation factors ranged from 1.09 to 4.00, with the highest values observed for acute upper respiratory tract infection (VIF 4.00) and the anti-infective treatment pattern (VIF 3.34), indicating no evidence of severe multicollinearity.

In a sensitivity analysis restricted to the 536 prescriptions containing a single respiratory diagnosis code, the main findings were preserved. Moderate or major potential DDI prevalence remained substantially higher in COPD than in asthma (51.6% vs. 20.7%). In Model B, medication burden remained strongly associated with the outcome (OR 2.66, 95% CI 2.02–3.50), as did cardiovascular or metabolic co-medication (OR 29.23, 95% CI 7.67–111.30) and COPD compared with asthma (OR 5.52, 95% CI 1.77–17.21). Estimates for some smaller diagnostic groups were less precise after restriction of the analytical sample.

## 3. Discussion

This retrospective community pharmacy study characterized respiratory pharmacotherapy and potential DDI risk using one index respiratory prescription per patient. Four main findings emerged. First, the assigned respiratory diagnostic groups showed distinct therapeutic profiles, ranging from predominantly inhaled maintenance therapy in asthma and COPD to anti-infective and symptomatic treatment in acute respiratory conditions. Second, at least one moderate or major potential DDI was identified in 28.0% of index prescriptions, with the highest prevalence among the major diagnostic groups observed in COPD. Third, recurrent interaction pairs showed that potential DDI burden frequently arose at the interface between respiratory treatment and cardiovascular, metabolic, anti-infective, analgesic, and other co-medications. Finally, medication burden and cardiovascular or metabolic co-medication showed the strongest positive independent associations with moderate or major potential DDI risk, while Model B showed improved fit after inclusion of therapeutic-pattern variables. Together, these findings indicate that medication safety assessment in respiratory prescribing should consider both the respiratory regimen and the broader pharmacotherapeutic context.

### 3.1. Diagnostic–Therapeutic Patterns in Respiratory Prescriptions

Therapeutic class distributions differed substantially across the assigned respiratory diagnostic groups (Table 3), consistent with the strong associations observed in the bivariate analyses. Asthma prescriptions were characterized predominantly by inhaled and intranasal corticosteroids and LABAs, consistent with the central role of anti-inflammatory and long-acting bronchodilator therapy in asthma management [29]. In contrast, acute upper respiratory tract infections and acute bronchitis or lower respiratory tract infections showed high use of anti-infective agents together with analgesic, antipyretic, and other symptomatic treatments, in agreement with previous drug utilization research in acute respiratory tract infections [1].

The COPD or chronic obstructive respiratory disease group showed a more complex pharmacotherapeutic profile, combining LABAs, LAMA/SAMA, inhaled corticosteroids, and frequent cardiovascular or metabolic co-medication (Table 3). Such treatment complexity is clinically plausible because COPD commonly coexists with cardiovascular and metabolic diseases and frequently requires concurrent respiratory maintenance therapy and treatment of non-respiratory chronic conditions [4,30]. Previous community pharmacy studies have similarly demonstrated the value of prescription and medication review data for characterizing respiratory pharmacotherapy, identifying drug-related problems, and supporting inhaler technique, adherence, and therapeutic optimization in patients with asthma or COPD [2,10,11,28].

The particularly strong association between diagnostic group and anti-infective use was driven largely by the concentration of these agents in acute respiratory conditions. Although our study did not assess prescribing appropriateness, indication validity, or the clinical necessity of anti-infective treatment, this pattern remains relevant to community pharmacy practice. Previous studies have shown that community pharmacy data can characterize medication use and pharmacist interventions in acute respiratory tract infections, while pharmacists may contribute to symptom assessment, referral, and antimicrobial stewardship [1,12,31]. The observed treatment distributions should therefore be interpreted as real-world drug utilization patterns instead of evidence of appropriate or inappropriate prescribing.

### 3.2. Potential DDI Burden Across Respiratory Diagnostic Groups

As shown in Table 4, at least one moderate or major potential DDI was identified in 180 of 643 index prescriptions (28.0%), but the burden varied substantially across the assigned respiratory diagnostic groups. Among the major diagnostic groups, the highest prevalence was observed in COPD or chronic obstructive respiratory disease, where 52.5% of index prescriptions contained at least one moderate or major potential DDI. This pattern is consistent with the greater therapeutic complexity observed in the COPD group, which combined respiratory maintenance treatment with frequent cardiovascular or metabolic co-medication (Table 3).

The COPD-specific prevalence observed in our cohort was similar in magnitude to the 53.6% prevalence of potentially severe DDIs reported among nursing home residents receiving COPD medications [32]. In hospitalized patients with respiratory disorders, DDIs were identified in 55% of patients at admission and in 96% during hospitalization [33]. Conversely, the COSYCONET COPD cohort reported serious adverse drug combinations in 4.2% of patients and potentially clinically relevant unwanted combinations in 6.4% [34], while another study of hospitalized patients with COPD reported serious potential DDI prevalences ranging from 11% to 49%, depending on the interaction checker used [35]. As emphasized in our previous clinical review, such variability is expected because DDI estimates are strongly influenced by care setting, patient complexity, medication burden, interaction database coverage, and severity classification criteria [4]. Direct numerical comparisons between studies should therefore be made cautiously.

These differences should not, however, be interpreted as direct effects of the respiratory diagnoses themselves. Potential DDI occurrence depends on the number and pharmacological composition of the active substances recorded on the prescription, as well as on concomitant treatment for non-respiratory conditions. The COPD findings are particularly consistent with this interpretation, because the same diagnostic group showed frequent LABA, LAMA/SAMA, ICS/INCS, and cardiovascular or metabolic medication use (Table 3). Thus, the elevated DDI burden in COPD likely reflects the broader pharmacotherapeutic context in which respiratory treatment is prescribed more than the respiratory diagnosis alone.

Overall, the results identify COPD as a particularly interaction-prone prescribing context within this community pharmacy cohort. Nevertheless, diagnostic group prevalence provides only a broad measure of interaction burden. Examination of the recurrent major and moderate potential DDI pairs (Table 5 and Table 6) provides additional information on the specific combinations contributing to these medication safety signals.

### 3.3. Clinical Relevance of Recurrent Potential DDI Pairs

Among major potential DDIs, the most recurrent pair was fenofibrate with rosuvastatin (Table 5). This combination may be used in selected patients with mixed dyslipidemia or persistent hypertriglyceridemia, but both agents are associated with muscle toxicity, and concomitant use may increase the risk of myopathy. Case reports have described marked creatine kinase elevation and clinically relevant muscle injury during statin-fibrate treatment [36], while a recent disproportionality analysis of the U.S. Food and Drug Administration Adverse Event Reporting System identified musculoskeletal and gastrointestinal safety signals for the rosuvastatin-fenofibrate combination [37]. Identification of this pair should therefore prompt review of the indication for combination therapy, prescribed doses, renal and hepatic function, and the presence of muscle symptoms instead of automatic rejection of the regimen.

Clopidogrel with omeprazole was another recurrent major potential DDI in our dataset. Clopidogrel is a prodrug whose bioactivation partly depends on CYP2C19. Omeprazole inhibits this enzyme and can reduce formation of the active clopidogrel metabolite, thereby attenuating platelet inhibition [38]. Consistent with this mechanism, concomitant omeprazole use has been associated with markedly higher P2Y12 platelet reactivity in patients receiving clopidogrel after neurovascular stenting [39]. Regulatory guidance therefore discourages concomitant use of clopidogrel with omeprazole or esomeprazole and recommends considering other proton pump inhibitors when gastroprotection is required [38]. However, the clinical outcome evidence is less consistent. In the randomized trial by Bhatt et al., omeprazole reduced gastrointestinal events without a significant increase in cardiovascular events. Premature trial termination limited the ability to exclude a clinically meaningful cardiovascular effect [40]. More recent real-world data likewise continue to regard clopidogrel with omeprazole or esomeprazole as an avoidable combination when suitable gastroprotective alternatives are available [41]. Accordingly, detection of this pair at dispensing should prompt verification of the patient’s actual medication use and the indication for gastroprotection. When concomitant use is confirmed, the community pharmacist can discuss the interaction with the patient and, where appropriate, contact the prescriber to consider a proton pump inhibitor with lower interaction potential, such as pantoprazole, rather than discontinuing gastroprotection altogether. Such prescription-level alerts illustrate the added value of pharmacist-mediated clinical verification because electronic dispensing data alone cannot establish whether all recorded medicines are actually used concomitantly by the patient.

The clarithromycin–fluticasone pair represents a pharmacokinetic interaction directly involving respiratory therapy. Clarithromycin is a strong CYP3A4 inhibitor, whereas fluticasone undergoes extensive CYP3A4-mediated metabolism. Inhibition of fluticasone metabolism may increase systemic corticosteroid exposure and the risk of corticosteroid-related adverse effects [42]. Beclomethasone has been proposed as a lower-interaction-risk inhaled corticosteroid when sustained treatment with a strong CYP3A4 inhibitor is unavoidable [42]. The clinical plausibility of this mechanism is supported by a case report of iatrogenic Cushing syndrome in a patient receiving inhaled fluticasone together with clarithromycin and itraconazole [43]. Because more than one CYP3A4 inhibitor was present in that case, the individual contribution of clarithromycin could not be isolated, but the report illustrates the potential consequences of combining fluticasone with strong metabolic inhibitors.

The recurrent dextromethorphan–tramadol pair is relevant because both drugs have serotonergic properties and may contribute to serotonin toxicity, particularly in the presence of additional serotonergic medications [23]. A published case described serotonin syndrome after dextromethorphan was added to a regimen containing tramadol and several antidepressants [44]. Although this event involved multiple serotonergic agents, it illustrates the importance of reviewing chronic medication before recommending or dispensing antitussive treatment.

The recurrent theophylline-beta-blocker alerts also require clinical contextualization. Beta-blockers differ substantially in beta-1 selectivity, dose-related pulmonary effects, and suitability in patients with obstructive airway disease [4]. Clinical studies in patients with COPD and cardiovascular disease have reported less favorable pulmonary effects with carvedilol than with the more beta-1 selective bisoprolol [45,46]. These findings support the pharmacological plausibility of the alerts identified in our dataset but do not constitute direct evidence of adverse outcomes caused by the specific theophylline-beta-blocker combinations.

Among moderate potential DDIs, betamethasone with ibuprofen was the most recurrent pair (Table 6). Concomitant exposure to systemic corticosteroids and NSAIDs may increase gastrointestinal toxicity. In a large study of patients with upper gastrointestinal bleeding, combined exposure to a non-selective NSAID and a corticosteroid was associated with particularly high gastrointestinal risk [47]. In our dataset, the recurrence of this pair supports assessment of treatment duration, previous gastrointestinal disease, age, concomitant antithrombotic therapy, and the potential need for gastroprotection.

Diuretic-containing recurrent pairs highlighted two opposing electrolyte-related risks at the interface between cardiovascular and respiratory pharmacotherapy. The combinations of indapamide with salmeterol or formoterol were classified as moderate potential DDIs. Indapamide may promote renal potassium loss, whereas beta-2 agonists shift potassium into cells, creating the potential for additive hypokalaemia and associated electrocardiographic effects [48,49]. Reviews addressing cardiovascular treatment in COPD further indicate that potassium-losing diuretics may cause dose-dependent hypokalaemia and metabolic alkalosis, potentially worsening carbon dioxide retention and ventilatory impairment, particularly when combined with beta-adrenergic agonists or systemic corticosteroids [50,51].

Conversely, spironolactone combinations with perindopril and candesartan, identified among the recurrent major potential DDIs (Table 5), reflect the risk of additive impairment of renal potassium elimination and hyperkalemia. A Romanian inpatient study similarly identified spironolactone-perindopril and spironolactone-candesartan among major potential DDIs [52]. Real-world evidence has shown an increased risk of hyperkalemia when spironolactone is combined with long-term angiotensin-converting enzyme (ACE) or angiotensin-receptor-blocker therapy [53], and hyperkalemia may occur even with low-dose spironolactone and candesartan [54]. Severe cases have also been reported during concomitant treatment with spironolactone and renin–angiotensin system inhibitors [55]. These combinations are not necessarily inappropriate but warrant review of indication and dose together with monitoring of serum potassium, renal function, and volume status.

The recurrent pairs in Table 5 and Table 6 signify priorities for targeted medication review, not evidence of inappropriate prescribing or confirmed adverse drug events. Their clinical relevance depends on indication, dose, treatment duration, comorbidities, laboratory findings, concurrent medications, and the feasibility of appropriate monitoring.

### 3.4. Interpretation of the Multivariable Model

Model B showed better statistical fit than Model A, indicating that therapeutic pattern and co-medication variables captured additional variation in potential DDI occurrence beyond demographics, diagnostic group, and medication burden (Table 7). Because these variables were derived from the same prescription composition used to determine DDI status, this improvement should be interpreted as descriptive and associative, without implying independent causal or predictive information.

Medication burden remained strongly associated with the presence of at least one moderate or major potential DDI after multivariable adjustment, with each additional active substance associated with approximately 2.4-fold higher odds of the outcome (OR 2.42, 95% CI 1.96–3.00). This finding is consistent with studies conducted in respiratory populations. Among pulmonary inpatients, the use of more than five drugs and the presence of comorbidities were independently associated with potential DDI exposure, whereas regimens containing five or fewer drugs were associated with substantially lower adjusted odds of an interaction [56]. Similarly, patients with severe asthma receiving biological therapy used an average of 10.4 medications and showed a high prevalence of potentially clinically significant DDIs, illustrating how treatment of respiratory disease and its comorbidities jointly contributes to medication complexity [57].

The association between medication burden and potential DDI risk also has a structural component. For a prescription containing *n* active substances, the number of possible pairwise combinations is n(n−1)/2. Consequently, the number of opportunities for potential interactions increases more rapidly than the medication count itself, even before the pharmacological properties of the individual substances are considered. Only a subset of these combinations represents clinically relevant interactions, but increasing regimen complexity substantially enlarges the interaction screening space [14,15,17].

Cardiovascular or metabolic co-medication showed the strongest positive class-level association in Model B (OR 14.16, 95% CI 5.25–38.21; Table 7). This broad operational category included several pharmacological classes with recognized interaction potential, including antithrombotics, beta blockers, calcium channel blockers, diuretics, lipid-lowering drugs, and antidiabetic agents. In older adults admitted with cardiovascular disease, polypharmacy was highly prevalent, and 77.5% had at least one severe potential DDI; clinically relevant combinations included non-selective beta blockers with beta-2 agonists, directly illustrating the interface between cardiovascular and respiratory pharmacotherapy [58]. Another cardiovascular inpatient study reported potentially relevant DDIs in 83.9% of patients, with occurrence increasing with medication number and multimorbidity and being particularly frequent among patients with concomitant respiratory disease [59]. At the population level, respiratory disease, including COPD, has also been shown to cluster with cardiometabolic conditions and their treatments, including antithrombotic and antihypertensive agents [60]. In severe asthma, commonly identified interactions have additionally involved antidiabetic drugs or diuretics combined with systemic corticosteroids [57]. These findings support interpreting cardiovascular or metabolic co-medication as a marker of both multimorbidity and exposure to pharmacologically complex regimens rather than as a homogeneous causal drug class. The wide confidence interval around our estimate further supports a cautious interpretation of its magnitude.

Several diagnostic group associations remained significant after adjustment. Compared with asthma, higher adjusted odds were observed for other chronic or unspecified respiratory or ENT conditions, acute upper respiratory tract infections, COPD or chronic obstructive respiratory disease, and acute bronchitis or lower respiratory tract infections (Table 7). Some of these coefficients differed markedly from the corresponding crude group differences and therefore require a conditional interpretation. This was particularly evident for acute upper respiratory tract infections: although the crude prevalence of moderate or major potential DDIs was similar to that observed in asthma (24.4% vs. 20.5%), the adjusted OR for acute upper respiratory tract infections was 7.60, while the anti-infective treatment indicator included in the same model showed an inverse association (OR 0.44). Because anti-infective treatment was strongly concentrated in acute respiratory conditions, simultaneous adjustment for diagnosis, medication burden, and therapeutic patterns changes the comparison represented by each coefficient. Accordingly, the diagnostic ORs should be interpreted as conditional associations within the fitted model, not as marginal differences in DDI prevalence or independent causal effects [61].

The inverse association between age ≥65 years and potential DDI risk (OR 0.30, 95% CI 0.13–0.68) also requires cautious interpretation. A large Swedish register study of adults aged ≥75 years similarly found that the probability of potentially serious DDIs decreased with increasing age after adjustment for medication count and sex, although the authors emphasized that this observation required further investigation [62]. In another population, the association between age and potential DDI risk was markedly attenuated after adjustment for non-disease-specific medication count and cardiovascular therapy, suggesting that regimen composition may explain part of the apparent age effect [63]. In our model, the age coefficient was estimated after simultaneous adjustment for medication burden, assigned diagnostic group, and therapeutic patterns, and should therefore not be interpreted as evidence that older patients are intrinsically protected against potential DDIs. Differences in regimen composition, prescribing practices, and the distribution of highly interacting combinations across age groups may have contributed to this result [64].

The inverse associations observed for the mucolytic/antitussive/expectorant pattern (OR 0.41, 95% CI 0.20–0.83) and LAMA/SAMA use (OR 0.29, 95% CI 0.09–0.98) should likewise not be interpreted as protective pharmacological effects. The former may identify prescriptions oriented toward short-term symptomatic treatment rather than complex chronic regimens, although individual agents within this heterogeneous category may still participate in clinically relevant interactions, as illustrated by the dextromethorphan–tramadol pair discussed above. For inhaled muscarinic antagonists, relatively limited systemic exposure may reduce their direct contribution to some systemic pharmacokinetic interactions [65,66]; however, the observed coefficient may also reflect their close association with specific COPD treatment profiles and the interdependence of diagnostic, therapeutic, and medication burden variables in the adjusted model.

### 3.5. Implications for Community Pharmacy Medication Safety

Beyond characterizing respiratory drug utilization patterns, community pharmacy prescription data can provide a useful basis for medication safety surveillance. Prescription processing and electronic decision support systems allow potential DDIs and other drug-related problems to be identified before dispensing, although the number and severity of generated alerts depend substantially on the interaction database and screening system used [13,19,21,67]. Romanian community pharmacy studies have likewise demonstrated a substantial burden of potential DDIs in routine outpatient prescriptions and among patients receiving statin therapy [14,15].

The present findings suggest several practical priorities for community pharmacy medication review. First, respiratory prescriptions containing a larger number of active substances may warrant greater attention, given the strong association between medication burden and moderate or major potential DDI risk in the multivariable analysis (Table 7). Second, respiratory treatment should not be assessed in isolation from concomitant therapy. Cardiovascular or metabolic co-medication showed the strongest positive class-level association with the outcome, supporting review of the broader pharmacotherapeutic context, particularly when respiratory and chronic cardiovascular or metabolic treatments coexist. COPD may represent an especially relevant setting for such review because this group combined substantial potential DDI burden with frequent comorbidity-related medication exposure (Table 3 and Table 4). The recurrent major and moderate potential DDI pairs identified in this study further illustrate specific combinations that may merit targeted assessment during dispensing (Table 5 and Table 6).

Potential DDI detection should be distinguished from pharmacovigilance reporting. An interaction alert identified during dispensing does not represent an adverse drug reaction but offers an opportunity for preventive medication review before clinical harm occurs. If a suspected adverse reaction is identified, Romanian community pharmacists can report it directly to the National Agency for Medicines and Medical Devices of Romania (ANMDMR) through the national spontaneous reporting system [68]. ANMDMR evaluates the report and then transmits it electronically to EudraVigilance, the European database for suspected adverse drug reactions. These reports contribute to ongoing drug safety monitoring [69].

The multivariable model should not be viewed as a substitute for pairwise DDI screening. Its value in the present study is primarily analytical, as it quantifies which broad prescription characteristics are associated with a higher concentration of potential DDI burden and helps identify prescribing contexts that may warrant closer medication review.

Electronic DDI alerts nevertheless require clinical interpretation. Interaction databases differ in coverage and severity classification, and excessive or poorly prioritized alerts may contribute to alert fatigue [19,20,21,67]. Prescription-level screening should therefore aim not to maximize the number of alerts but to identify potential DDIs that are most relevant to the individual therapeutic context. Factors such as medication burden, interacting drug classes, treatment indication, dose, treatment duration, comorbidities, and available clinical or laboratory information should inform this assessment.

### 3.6. Study Limitations

Several limitations should be considered. The retrospective observational design precludes causal inference, and data from only two community pharmacies in one Romanian urban area may limit generalizability. Because no common patient identifier was available across providers, residual duplicate representation across the two provider datasets cannot be completely excluded, although no exact cross-provider profile matches were identified.

Because prescriptions containing a single active substance were not represented in the source dataset, the reported potential DDI prevalence applies to respiratory prescriptions containing at least two active substances.

Because the source dataset retained only the earliest prescription by dispensing date for each patient before the respiratory-specific analysis, subsequent prescriptions were unavailable. The study, therefore, did not capture longitudinal treatment changes, refill persistence, within-patient seasonal variation, cumulative medication exposure, or alternative prescription selection strategies. The retained prescription also did not necessarily represent the patient’s complete concurrent medication regimen. Information on dose, treatment duration, adherence, renal or hepatic function, laboratory findings, and clinical outcomes was unavailable; consequently, identified DDIs represent potential prescription-level interactions rather than confirmed adverse events.

DDI identification depended on DrugBank coverage and severity classification, and results may differ from those of other interaction databases. Diagnostic grouping was based on the first eligible respiratory code when multiple codes were present, which simplified more complex diagnostic profiles. Several diagnostic subgroups were also small, leading to imprecise estimates and wide confidence intervals. The main findings were preserved in the sensitivity analysis restricted to prescriptions containing a single respiratory diagnostic code.

The multivariable model also has a structural limitation, as both the outcome and several predictors are derived from the same prescription’s medication composition. Potential DDIs were identified based on active substance combinations, whereas medication burden and therapeutic class indicators summarized that same prescription. Consequently, some associations, particularly those involving cardiovascular or metabolic co-medication, may partly reflect the pharmacological composition that generates the DDI outcome itself and should not be interpreted as independent epidemiological effects.

## 4. Materials and Methods

### 4.1. Study Design, Data Source, and Ethical Considerations

We conducted a retrospective, observational, and non-interventional study based on the secondary analysis of anonymized electronic prescription data from two community pharmacies in Timișoara, Romania, covering the period from November 2022 to October 2023. The data originated from routine electronic prescriptions processed during dispensing and reimbursement within the Romanian national health insurance system and were not collected specifically for the present study. The two pharmacies were purposively selected as collaborating sites based on their willingness to provide anonymized routine electronic dispensing data for academic research; no probabilistic sampling of pharmacies was performed. The source dataset had originally been assembled for drug–drug interaction analysis and therefore retained prescriptions containing at least two active substances; it covered a broad range of medical conditions.

The source prescription databases were maintained separately by the two data providers. Patient identifiers available exclusively within each provider’s information system were used before anonymization to identify repeated prescriptions. Details of patient-level record retention, anonymization, dataset combination, and cohort derivation are provided in Section 4.2.

The anonymized datasets contained only variables required for the study objectives, including age, sex, diagnosis code or codes, dispensed medicinal products, and dispensing date. These variables were extracted from structured fields in the national electronic prescribing and dispensing system and were not manually transcribed from paper prescriptions. Completeness was assessed at the record level during cohort derivation. The research team had no access to the identifiable source databases, direct patient identifiers, or re-identification keys.

The secondary analysis of the anonymized data was approved by the Scientific Research Ethics Committee of the Victor Babeș University of Medicine and Pharmacy Timișoara, Romania (Approval No. 62/23 July 2026). Under the approved retrospective and non-interventional protocol, individual informed consent was not required because no participants were recruited or contacted, no intervention or modification of treatment was undertaken, and the research team had access only to anonymized data. The study was conducted in accordance with the principles of the Declaration of Helsinki and applicable institutional requirements concerning research integrity, confidentiality, and data protection.

### 4.2. Cohort Derivation and Analytical Unit

Within each provider database, repeated prescriptions belonging to the same patient were identified using patient identifiers available exclusively to that provider. During the 12-month observation period, only the earliest prescription by dispensing date was retained for each patient, irrespective of diagnosis; subsequent prescriptions from the same patient were not included. This patient-level retention procedure was performed before anonymization and before the present respiratory analysis.

After anonymization, the two provider-level datasets were combined. Because no common patient identifier was available across providers, potential duplicate representation was assessed using exact profile matching based on age, sex, the complete sequence of diagnosis codes, and the complete recorded therapeutic regimen. No exact cross-provider matches were identified. Because exact profile matching cannot establish patient identity across providers, residual duplicate representation cannot be completely excluded.

The resulting source dataset comprised 3300 combined, deduplicated, and anonymized prescription records containing at least two active substances, with one prescription record retained per patient within each provider-level dataset. From this source dataset, we selected records containing at least one diagnosis code within the predefined respiratory range—codes 498–542 of the Romanian 999-code disease list. The Romanian 999-code list is aligned with the International Classification of Diseases, 10th Revision [70]. The respiratory codes included in the study, their corresponding diagnostic labels, and analytical group assignments are provided in Supplementary Table S1.

Of the 3300 source records, 661 contained at least one eligible respiratory diagnosis code. Eighteen records with missing age and/or sex were excluded, yielding a final complete case analytical cohort of 643 patients, each represented by one respiratory prescription.

The analytical dataset contained one retained respiratory prescription per patient. Accordingly, demographic characteristics were interpreted at the patient level, whereas medication use, therapeutic patterns, and potential DDIs were interpreted at the level of the retained prescription. For consistency throughout the manuscript, this prescription is referred to as the index respiratory prescription. Because all eligible records available in the source dataset for the predefined observation period were included, no formal a priori sample size calculation was performed.

### 4.3. Diagnostic Grouping, Medication Standardization and Therapeutic Class Assignment

For each index prescription, respiratory diagnosis codes were mapped to clinically interpretable diagnostic groups. When more than one respiratory diagnosis code was present, the first recorded respiratory code was used to define the main diagnostic group for the mutually exclusive group-based analyses. The final categories were asthma, acute upper respiratory tract infections, chronic obstructive pulmonary disease (COPD) or chronic obstructive respiratory disease, acute bronchitis or lower respiratory tract infections, allergic rhinitis or chronic rhinosinusitis, other chronic or unspecified respiratory or ear, nose, and throat (ENT) conditions, influenza or pneumonia, and other respiratory conditions. The underlying Romanian diagnostic codes, corresponding diagnostic labels, and analytical group assignments are provided in Supplementary Table S1.

Romanian medicinal product and active substance names were standardized to the corresponding English active substance names used in DrugBank [48] to ensure compatibility with potential DDI screening. Fixed-dose combination products were decomposed into their individual active substances; consequently, a two- or three-component combination contributed two or three active substances, respectively, to medication burden calculations, therapeutic class assignment, and DDI assessment.

Each standardized active substance was then assigned to one or more predefined operational therapeutic classes according to its pharmacological and clinical role. The classes used in the analyses were inhaled and intranasal corticosteroids (ICS/INCS), long-acting beta-2 agonists (LABA), short-acting beta-2 agonists (SABA), long- and short-acting muscarinic antagonists (LAMA/SAMA), antileukotrienes, anti-infectives, antihistamine and ear, nose, and throat (ENT) preparations, mucolytic, antitussive, and expectorant agents, systemic corticosteroids, non-steroidal anti-inflammatory drug (NSAID), analgesic, and antipyretic agents, gastroprotective drugs, and cardiovascular or metabolic drugs. These categories were operational groupings created for the present analysis and were used consistently for descriptive, bivariate, and multivariable analyses.

### 4.4. Medication Burden and Drug–Drug Interaction Assessment

We defined medication burden as the number of standardized active substances recorded on the index respiratory prescription. For fixed-dose combination products, each active component was counted separately. Medication burden was summarized descriptively using mean, standard deviation, median, and interquartile range. For categorical analyses, prescriptions were grouped into three predefined categories: two active substances, three to four active substances, and five or more active substances. The threshold of five or more active substances was used as the operational definition of polypharmacy [17]. In the multivariable analysis of moderate or major potential DDI risk, medication burden was modeled as a continuous variable representing the number of active substances per index prescription.

Potential DDI assessment was performed at the index prescription level after medication standardization. All standardized active substances recorded on each index prescription were screened pairwise using DrugBank Online and its application programming interface, version 5.1.11 [48]. Interactions returned by DrugBank were classified as minor, moderate, or major according to the severity assigned in the database, whereas pairs for which no interaction was returned were recorded as “no interaction found.” For each index prescription, the numbers of moderate and major potential DDIs were determined, together with the presence or absence of at least one moderate or major potential DDI. The latter binary variable constituted the primary DDI outcome used in the multivariable analyses. Minor interactions were not included in the primary outcome because the study focused on interaction categories with greater potential clinical relevance.

All identified interactions were interpreted as potential DDIs and not as confirmed clinically manifest interactions or adverse drug events, because the available data did not include clinical outcomes that would allow assessment of actual patient harm.

### 4.5. Descriptive Statistical Analysis

Continuous variables were summarized using mean and standard deviation (SD) and, where appropriate, median and interquartile range (IQR). Categorical variables were summarized as absolute frequencies and percentages. Descriptive analyses included demographic characteristics, diagnostic groups, medication burden, therapeutic classes, treatment patterns, and potential DDI outcomes.

Medication burden was summarized overall and by diagnostic group. The frequencies of prescriptions containing at least one moderate potential DDI, at least one major potential DDI, and at least one moderate or major potential DDI were calculated for the full analytical cohort and by diagnostic group. Because each patient contributed one retained index respiratory prescription, these prescription-level frequencies were numerically equivalent to patient-level prevalences within the analytical cohort.

### 4.6. Bivariate Analysis

Bivariate analyses were used to examine associations between assigned respiratory diagnostic groups, therapeutic classes, treatment patterns, medication burden, and potential DDI outcomes. Associations between diagnostic group and categorical variables were assessed using chi-square tests, with Cramer’s *V* reported as a measure of association strength. Continuous medication burden was compared across diagnostic groups using the Kruskal–Wallis test. For families of related bivariate tests, *p*-values were adjusted for multiple comparisons using the Benjamini–Hochberg false-discovery-rate procedure. Statistical significance was defined as a two-sided adjusted p<0.05 where multiple-testing correction was applied.

### 4.7. Multivariable Analysis

Multivariable logistic regression was used to identify factors independently associated with the presence of at least one moderate or major potential DDI at the index respiratory prescription. The binary dependent variable was coded as the presence versus absence of at least one moderate or major potential DDI.

We fitted two models. Model A included demographic characteristics, assigned respiratory diagnostic group, and medication burden expressed as the continuous number of active substances per index prescription. Model B extended Model A by incorporating selected therapeutic pattern indicators representing clinically relevant medication profiles: ICS/LABA therapy, anti-infective treatment, mucolytic, antitussive, and expectorant treatment, ENT/allergic treatment, cardiovascular or metabolic co-medication, and LAMA/SAMA use. Age was entered categorically as <18, 18–64, and ≥65 years, with 18–64 years as the reference category. Female sex and asthma were used as the reference categories for sex and diagnostic group, respectively. Multicollinearity among predictors included in Model B was assessed using variance inflation factors (VIFs).

For each fitted model, the log odds of the binary outcome were modeled as a linear combination of the included predictors. Adjusted odds ratios were obtained by exponentiating the multivariable logistic regression coefficients (aOR = $e^{\beta }$). Each aOR represents the association between a predictor and the presence of at least one moderate or major potential DDI while holding other covariates constant. For categorical predictors, aORs were calculated relative to the prespecified reference category. For medication burden, the aOR corresponds to a one-unit increase in the number of active substances. Ninety-five percent confidence intervals were calculated on the log odds scale and exponentiated to obtain the corresponding confidence intervals for the aORs. Corresponding two-sided *p*-values were reported. Model fit was compared using the Akaike information criterion (AIC), Bayesian information criterion (BIC), and McFadden’s pseudo-$R^{2}$.

As a sensitivity analysis, Models A and B were repeated after excluding prescriptions containing more than one distinct respiratory diagnosis code, thereby restricting the analysis to records containing a single respiratory diagnostic code.

Statistical analyses were performed in Python version 3.12.7 using pandas version 2.2.2, NumPy version 1.26.4, SciPy version 1.13.1, and statsmodels version 0.14.2.

## 5. Conclusions

This study shows that community pharmacy respiratory prescriptions can characterize real-world therapeutic patterns and identify potential medication safety signals. Moderate or major potential DDIs were present in 28.0% of index prescriptions, with the highest burden among the major diagnostic groups observed in COPD. Medication burden and cardiovascular or metabolic co-medication showed the strongest positive independent associations with potential DDI risk. These findings support targeted medication review in community pharmacy, particularly when respiratory treatment is combined with multiple active substances and comorbidity-related therapy. Potential DDI assessment should therefore consider not only the respiratory regimen itself but also the broader pharmacotherapeutic context.

## Figures and Tables

**Figure 1:**
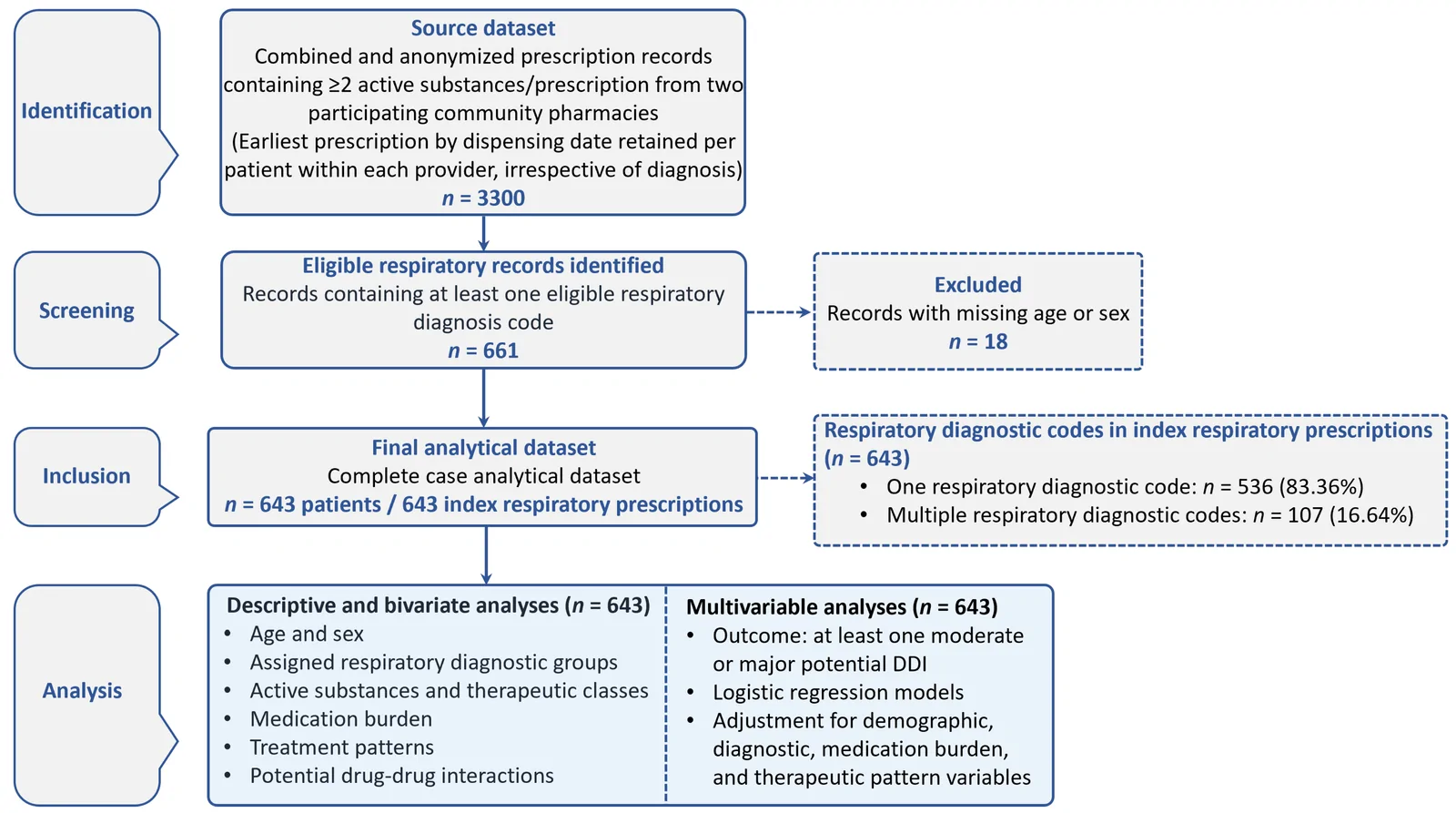
Study flow diagram showing derivation of the analytical dataset.

**Table 1:** Demographic and diagnostic characteristics of the analytical cohort represented by one index respiratory prescription per patient (N=643).

Characteristic	Value
**Age **	
Age, years, mean ± SD	46.26 ± 25.03
Age, years, median [IQR]	52 [23.5–66.0]
Age range, years	1–93
**Age group, n (%)**	
<18 years	139 (21.62)
18–64 years	326 (50.70)
≥65 years	178 (27.68)
**Sex, n (%)**	
Female	326 (50.70)
Male	317 (49.30)
**Number of distinct respiratory diagnostic codes on the index prescription, n (%)**	
One respiratory diagnostic code	536 (83.36)
Multiple respiratory diagnostic codes	107 (16.64)
**Assigned respiratory diagnostic group, n (%)**	
Asthma	264 (41.06)
Acute upper respiratory tract infections	127 (19.75)
COPD or chronic obstructive respiratory disease	99 (15.40)
Acute bronchitis or lower respiratory tract infections	57 (8.86)
Allergic rhinitis or chronic rhinosinusitis	43 (6.69)
Other chronic or unspecified respiratory or ENT conditions	32 (4.98)
Influenza or pneumonia	16 (2.49)
Other respiratory conditions	5 (0.78)

COPD, chronic obstructive pulmonary disease; ENT, ear, nose, and throat; IQR, interquartile range; SD, standard deviation.

**Table 2:** Medication burden and most frequent active substances recorded on the index respiratory prescription (N=643).

Variable	Value
**Medication burden**	
Number of active substances, mean ± SD	3.65 ± 2.07
Number of active substances, median [IQR]	3 [2–4]
Number of active substances, range	2–22
**Medication-burden category, n (%)**	
2 active substances	231 (35.93)
3–4 active substances	263 (40.90)
≥5 active substances	149 (23.17)
**Most frequent active substances, n (%)**	
Formoterol	163 (25.35)
Fluticasone	139 (21.62)
Budesonide	129 (20.06)
Salmeterol	119 (18.51)
Desloratadine	87 (13.53)
Amoxicillin	84 (13.06)
Ibuprofen	83 (12.91)
Clavulanic acid	79 (12.29)
Montelukast	58 (9.02)
Beclomethasone dipropionate	45 (7.00)
Betamethasone	42 (6.53)
Levocetirizine	37 (5.75)
Tetryzoline	35 (5.44)
Theophylline	35 (5.44)
Esomeprazole	35 (5.44)
Carbocysteine	33 (5.13)
Dextromethorphan	33 (5.13)
Mometasone	32 (4.98)
Salbutamol	32 (4.98)
Cefuroxime	31 (4.82)

Romanian medicinal product and active substance names were standardized to the corresponding English active substance names used in DrugBank. Fixed-dose combination products were decomposed into their individual active substances; therefore, active substance frequencies are not mutually exclusive. All clavulanic acid occurrences reflected amoxicillin/clavulanic acid combination products rather than standalone prescribing.

**Table 3:** Therapeutic classes recorded on the index respiratory prescription by diagnostic group. Values are shown as *n* (%) within each diagnostic group.

**Panel A. Respiratory Maintenance and Corticosteroid-Related Therapeutic Classes**						
**Diagnostic Group**	**ICS/INCS**	**LABA**	**SABA**	**LAMA/SAMA**	**LTRA**	**Systemic CS**
Asthma (n=264)	242 (91.7)	225 (85.2)	14 (5.3)	2 (0.8)	48 (18.2)	4 (1.5)
Acute URTI (n=127)	17 (13.4)	5 (3.9)	1 (0.8)	0 (0.0)	1 (0.8)	30 (23.6)
COPD/chronic obstructive disease (n=99)	42 (42.4)	54 (54.5)	6 (6.1)	38 (38.4)	3 (3.0)	1 (1.0)
Other chronic or unspecified respiratory/ENT conditions (n=32)	14 (43.8)	10 (31.2)	1 (3.1)	1 (3.1)	0 (0.0)	3 (9.4)
Acute bronchitis or lower RTI (n=57)	5 (8.8)	2 (3.5)	10 (17.5)	0 (0.0)	0 (0.0)	10 (17.5)
Allergic rhinitis or CRS (n=43)	21 (48.8)	2 (4.7)	0 (0.0)	0 (0.0)	4 (9.3)	3 (7.0)
Influenza/pneumonia (n=16)	6 (37.5)	2 (12.5)	0 (0.0)	0 (0.0)	2 (12.5)	2 (12.5)
Other respiratory conditions (n=5)	0 (0.0)	0 (0.0)	0 (0.0)	1 (20.0)	0 (0.0)	3 (60.0)
**Panel B. Anti-infective, symptomatic, gastroprotective, and comorbidity-related therapeutic classes**						
**Diagnostic Group**	**Anti-Infectives**	**Antihistamine and ENT Preparations**	**Mucolytic, Antitussive, or Expectorant Agents**	**NSAID, Analgesic, or Antipyretic Agents**	**Gastroprotective Drugs**	**Cardiovascular or Metabolic Drugs**
Asthma (n=264)	9 (3.4)	33 (12.5)	6 (2.3)	7 (2.7)	18 (6.8)	50 (18.9)
Acute URTI (n=127)	112 (88.2)	45 (35.4)	59 (46.5)	78 (61.4)	7 (5.5)	3 (2.4)
COPD/chronic obstructive disease (n=99)	6 (6.1)	6 (6.1)	11 (11.1)	8 (8.1)	14 (14.1)	52 (52.5)
Other chronic or unspecified respiratory/ENT conditions (n=32)	23 (71.9)	11 (34.4)	6 (18.8)	16 (50.0)	7 (21.9)	3 (9.4)
Acute bronchitis or lower RTI (n=57)	51 (89.5)	20 (35.1)	28 (49.1)	26 (45.6)	4 (7.0)	3 (5.3)
Allergic rhinitis or CRS (n=43)	9 (20.9)	38 (88.4)	3 (7.0)	15 (34.9)	9 (20.9)	16 (37.2)
Influenza/pneumonia (n=16)	14 (87.5)	7 (43.8)	6 (37.5)	8 (50.0)	8 (50.0)	4 (25.0)
Other respiratory conditions (n=5)	1 (20.0)	0 (0.0)	0 (0.0)	0 (0.0)	2 (40.0)	2 (40.0)

ICS/INCS, inhaled and intranasal corticosteroids; LABA, long-acting beta-2 agonists; SABA, short-acting beta-2 agonists; LAMA/SAMA, long-acting or short-acting muscarinic antagonists; LTRA, leukotriene receptor antagonists; CS, corticosteroids; URTI, upper respiratory tract infection; RTI, respiratory tract infection; CRS, chronic rhinosinusitis; COPD, chronic obstructive pulmonary disease; ENT, ear, nose, and throat; NSAID, non-steroidal anti-inflammatory drug. Therapeutic classes are not mutually exclusive because one index prescription could contain multiple active substances from different classes.

**Table 4:** Potential drug–drug interaction burden overall and by assigned respiratory diagnostic group. Values are shown as *n* (%) within each row.

Group	*N*	At Least One Moderate Potential DDI	At Least One Major Potential DDI	At Least One Moderate or Major Potential DDI
Overall analytical cohort	643	171 (26.59)	45 (7.00)	180 (27.99)
Asthma	264	49 (18.6)	17 (6.4)	54 (20.5)
Acute upper respiratory tract infections	127	31 (24.4)	5 (3.9)	31 (24.4)
COPD or chronic obstructive respiratory disease	99	48 (48.5)	18 (18.2)	52 (52.5)
Other chronic or unspecified respiratory or ENT conditions	32	11 (34.4)	2 (6.2)	11 (34.4)
Acute bronchitis or lower respiratory tract infections	57	14 (24.6)	0 (0.0)	14 (24.6)
Allergic rhinitis or chronic rhinosinusitis	43	10 (23.3)	1 (2.3)	10 (23.3)
Influenza or pneumonia	16	6 (37.5)	2 (12.5)	6 (37.5)
Other respiratory conditions	5	2 (40.0)	0 (0.0)	2 (40.0)

COPD, chronic obstructive pulmonary disease; DDI, drug–drug interaction; ENT, ear, nose, and throat. Moderate and major potential DDIs were not mutually exclusive because the same index prescription could contain both severity categories. The combined outcome represents the presence of at least one moderate or major potential DDI on the index respiratory prescription.

**Table 5:** Recurrent major potential drug–drug interaction pairs detected on the index respiratory prescription. Percentages were calculated using the full analytical cohort as denominator (N=643). The table includes major potential DDI pairs observed in two or more index prescriptions.

Major Potential DDI Pair	Index Prescriptions, *n*	Percentage of Total Cohort (%)
Fenofibrate + rosuvastatin	7	1.09
Clopidogrel + omeprazole	4	0.62
Bisoprolol + theophylline	3	0.47
Candesartan + spironolactone	3	0.47
Perindopril + spironolactone	3	0.47
Amlodipine + simvastatin	2	0.31
Atorvastatin + fenofibrate	2	0.31
Carvedilol + theophylline	2	0.31
Clarithromycin + fluticasone	2	0.31
Codeine + furazolidone	2	0.31
Dextromethorphan + tramadol	2	0.31
Nebivolol + theophylline	2	0.31

**Table 6:** Top recurrent moderate potential drug–drug interaction pairs detected on the index respiratory prescription. Percentages were calculated using the full analytical cohort as denominator (N=643). The table reports the ten most frequent moderate potential DDI pairs.

Moderate Potential DDI Pair	Index Prescriptions, *n*	Percentage of Total Cohort (%)
Betamethasone + ibuprofen	22	3.42
Indapamide + perindopril	11	1.71
Bisoprolol + indapamide	8	1.24
Budesonide + nebivolol	7	1.09
Indapamide + salmeterol	7	1.09
Formoterol + indapamide	6	0.93
Formoterol + nebivolol	6	0.93
Salbutamol + salmeterol	6	0.93
Budesonide + indapamide	5	0.78
Budesonide + perindopril	5	0.78

**Table 7:** Multivariable logistic regression results for moderate or major potential drug–drug interaction risk at the index respiratory prescription. Odds ratios are shown for Model B. Model fit measures are shown for Model A and Model B.

Predictor	Adjusted OR (95% CI)	*p*-Value
**Demographic variables**		
Age <18 years vs. 18–64 years	1.09 (0.50–2.34)	0.835
Age ≥65 years vs. 18–64 years	0.30 (0.13–0.68)	0.004
Male vs. female	1.36 (0.80–2.31)	0.250
**Diagnostic group**		
Other respiratory conditions vs. asthma	2.14 (0.19–24.12)	0.538
Other chronic or unspecified respiratory or ENT conditions vs. asthma	9.80 (2.80–34.37)	<0.001
COPD or chronic obstructive respiratory disease vs. asthma	5.31 (2.04–13.86)	<0.001
Acute bronchitis or lower respiratory tract infections vs. asthma	5.26 (1.32–20.88)	0.018
Influenza or pneumonia vs. asthma	1.78 (0.20–15.78)	0.606
Acute upper respiratory tract infections vs. asthma	7.60 (2.21–26.04)	0.001
Allergic rhinitis or chronic rhinosinusitis vs. asthma	0.56 (0.15–2.04)	0.379
**Medication burden and therapeutic patterns**		
Number of active substances, per additional substance	2.42 (1.96–3.00)	<0.001
ICS/LABA pattern	1.91 (0.79–4.57)	0.148
Anti-infective treatment pattern	0.44 (0.18–1.08)	0.074
Mucolytic/antitussive/expectorant pattern	0.41 (0.20–0.83)	0.013
ENT/allergic treatment pattern	0.89 (0.45–1.77)	0.745
Cardiovascular/metabolic co-medication	14.16 (5.25–38.21)	<0.001
LAMA/SAMA use	0.29 (0.09–0.98)	0.046
**Model fit**		
	**Model A**	**Model B**
*N*	643	643
AIC	498.24	442.71
BIC	551.83	523.10
McFadden pseudo-$R^{2}$	0.378	0.467

OR, odds ratio; CI, confidence interval; DDI, drug–drug interaction; ENT, ear, nose, and throat; COPD, chronic obstructive pulmonary disease; ICS/LABA, inhaled corticosteroid/long-acting beta_2_-agonist treatment pattern; LAMA/SAMA, long-acting or short-acting muscarinic antagonist; AIC, Akaike information criterion; BIC, Bayesian information criterion.

## Data Availability

The data presented in this study are available upon request from the corresponding author due to the ethical restrictions. Aggregated data supporting the reported results are presented in the article and Supplementary Materials.

## Supplementary Materials

The following supporting information can be downloaded at https://www.mdpi.com/article/10.3390/ph19101528/s1: Table S1: Respiratory diagnostic codes from the Romanian 999-code disease list, corresponding diagnostic labels, and assigned analytical diagnostic groups; Table S2: Medication burden by assigned respiratory diagnostic group; Table S3: Multivariable logistic regression results for Model A examining factors associated with at least one moderate or major potential drug–drug interaction at the index respiratory prescription.

## Abbreviations

The following abbreviations are used in this manuscript:

AIC	Akaike information criterion
ANMDMR	National Agency for Medicines and Medical Devices of Romania
BIC	Bayesian information criterion
COPD	Chronic obstructive pulmonary disease
CRS	Chronic rhinosinusitis
CS	Corticosteroids
DDI	Drug–drug interaction
ENT	Ear, nose, and throat
ICS	Inhaled corticosteroids
INCS	Intranasal corticosteroids
LABA	Long-acting beta-2 agonists
LAMA	Long-acting muscarinic antagonists
LTRA	Leukotriene receptor antagonists
NSAID	Non-steroidal anti-inflammatory drug
RTI	Respiratory tract infection
SABA	Short-acting beta-2 agonists
SAMA	Short-acting muscarinic antagonists
URTI	Upper respiratory tract infection
VIF	Variance inflation factor
