# Supplementary material for: Drug Utilization Patterns and Potential Drug–Drug Interaction Burden in Respiratory Prescriptions: A Real-World Community Pharmacy Study
Source: Pharmaceuticals (Basel). 2026 Sep 26;19(10):1528. doi: 10.3390/ph19101528 (PMC13708138; doi:10.3390/ph19101528)
Supplement: Supplementary file 1 [file pharmaceuticals-19-01528-s001.zip › pharmaceuticals-4529788-supplementary.pdf]

Supplementary Table S1. Respiratory diagnostic codes from the Romanian 999-code disease list, corresponding diagnostic labels, and assigned analytical diagnostic groups.

Romanian 999-code	Diagnostic label (Romanian)	Diagnostic label (English)	Assigned analytical diagnostic group
498	Rino-faringita acuta [guturaiul comun]	Acute nasopharyngitis (common cold)	Acute upper respiratory tract infections
499	Sinuzita acuta	Acute sinusitis	Acute upper respiratory tract infections
500	Faringita si amigdalita acuta	Acute pharyngitis and tonsillitis	Acute upper respiratory tract infections
501	Laringita si traheita acuta	Acute laryngitis and tracheitis	Acute upper respiratory tract infections
502	Laringita obstruct ac(crup) si epiglotita ac	Acute obstructive laryngitis (croup) and epiglottitis	Acute upper respiratory tract infections
503	I.C.R.S cu loc.multiple si neprecizate	Acute respiratory infections, multiple and unspecified sites	Acute upper respiratory tract infections
504	Gripa, cu virus gripal identificat	Influenza with identified influenza virus	Influenza or pneumonia
505	Gripa, cu virus neidentificat	Influenza with unidentified virus	Influenza or pneumonia
506	Pneumonia virala, neclasata la alte locuri	Viral pneumonia, not classified elsewhere	Influenza or pneumonia
507	Pneumonia prin Streptococcus pneumoniae	Pneumonia due to Streptococcus pneumoniae	Influenza or pneumonia
508	Pneumonia prin Haemophilus influenzae	Pneumonia due to Haemophilus influenzae	Influenza or pneumonia
509	Pneumonii bacteriene, neclas la alte locuri	Other bacterial pneumonias, not classified elsewhere	Influenza or pneumonia
510	Pneumonia datorita altor microorg infect.	Pneumonia due to other infectious organisms	Influenza or pneumonia
511	Pneumonii cu micro-organisme neprecizate	Pneumonias with unspecified microorganisms	Influenza or pneumonia
512	Bronsita si bronsiolita acuta	Acute bronchitis and bronchiolitis	Acute bronchitis or lower respiratory tract infections
513	Inf ac ale cailor resp inferioare, fara prec	Lower respiratory infections, unspecified	Acute bronchitis or lower respiratory tract infections
514	Rinita alergica si vazomotorie	Allergic and vasomotor rhinitis	Allergic rhinitis or chronic rhinosinusitis
515	Rinita, rino-faringita si faringita cronica	Chronic rhinitis, nasopharyngitis, and pharyngitis	Other chronic or unspecified respiratory or ENT conditions
516	Sinuzita cronica	Chronic sinusitis	Allergic rhinitis or chronic rhinosinusitis
517	Boli cronice ale amigdale si veget. adenoide	Chronic diseases of tonsils and adenoids	Other chronic or unspecified respiratory or ENT conditions
518	Angina flegmonoasa	Peritonsillar abscess (quinsy)	Other chronic or unspecified respiratory or ENT conditions
519	Laringita si laringo-traheita	Laryngitis and laryngotracheitis	Other chronic or unspecified respiratory or ENT conditions
520	Boli corzi vocale si ale laringe, neclas	Diseases of vocal cords and larynx, unspecified	Other chronic or unspecified respiratory or ENT conditions

521	Alte boli ale cailor respir. superioare	Other diseases of upper respiratory tract	Other chronic or unspecified respiratory or ENT conditions
522	Bronsita neprecizata ca acuta sau cronica	Bronchitis, unspecified as acute or chronic	Other chronic or unspecified respiratory or ENT conditions
523	Bronsita cronica simpla si mucopurulenta	Simple and mucopurulent chronic bronchitis	COPD or chronic obstructive respiratory disease
524	Bronsita cronica FAI	Chronic obstructive pulmonary disease	COPD or chronic obstructive respiratory disease
525	Emfizemul	Emphysema	COPD or chronic obstructive respiratory disease
526	Alte boli pulmonare obstructive cronice	Other chronic obstructive pulmonary diseases	COPD or chronic obstructive respiratory disease
527	Astmul	Asthma	Asthma
528	Bronsiectazia	Bronchiectasis	Other respiratory conditions
529	Pneumoconioza de carbune, a minerilor	Coal worker's pneumoconiosis	Other respiratory conditions
530	Pneumoconioza dat. azbest, altor fibre minerale	Asbestosis and other pneumoconioses due to mineral fibers	Other respiratory conditions
531	Pneumoconioza datorita pulberii de siliciu	Silicosis and other pneumoconioses due to dust	Other respiratory conditions
532	Pneumoconioza dat. altor pulberi neorganice	Pneumoconiosis due to other inorganic dusts	Other respiratory conditions
533	Pneumoconioza asociata cu tuberculoza	Pneumoconiosis associated with tuberculosis	Other respiratory conditions
534	Afec ale cai aeriene dat pulberilor organ preciz	Airway diseases due to organic dust	Other respiratory conditions
535	Afec respiratorii datorita altor agenti externi	Respiratory diseases due to other external agents	Other respiratory conditions
536	Edem pulmonar	Pulmonary edema	Other respiratory conditions
537	Alte afectiuni pulmonare interstitiale	Other interstitial pulmonary diseases	Other respiratory conditions
538	Abcesul pulmonar si al mediastinului	Lung and mediastinal abscess	Other respiratory conditions
539	Pneumothorax	Pneumothorax	Other respiratory conditions
540	Afectiuni pleurale	Pleural diseases	Other respiratory conditions
541	Tulb.resp.dupa un act medic priv.diag. si trat.	Respiratory disorders following medical procedures for diagnosis and treatment	Other respiratory conditions
542	Alte tulburari respiratorii	Other respiratory disorders	Other respiratory conditions

Supplementary Table S2. Medication burden by assigned respiratory diagnostic group.

Assigned respiratory diagnostic group	*N*	Active substances, mean ± SD	Median [IQR]	2 active substances, *n* (%)	3–4 active substances, *n* (%)	≥5 active substances, *n* (%)
Asthma	264	3.22 ± 2.42	2 [2–3]	159 (60.2)	59 (22.3)	46 (17.4)
Acute upper respiratory tract infections	127	3.56 ± 1.34	3 [3–4]	25 (19.7)	82 (64.6)	20 (15.7)
COPD or chronic obstructive respiratory disease	99	4.26 ± 1.96	4 [3–6]	23 (23.2)	37 (37.4)	39 (39.4)
Acute bronchitis or lower respiratory tract infections	57	3.96 ± 1.52	4 [3–5]	9 (15.8)	31 (54.4)	17 (29.8)
Allergic rhinitis or chronic rhinosinusitis	43	4.05 ± 1.65	4 [3–4.5]	6 (14.0)	26 (60.5)	11 (25.6)
Other chronic or unspecified respiratory or ENT conditions	32	3.69 ± 1.42	4 [3–4]	6 (18.8)	20 (62.5)	6 (18.8)
Influenza or pneumonia	16	5.63 ± 3.12	4.5 [3–7]	1 (6.3)	7 (43.8)	8 (50.0)
Other respiratory conditions	5	3.60 ± 1.52	4 [2–5]	2 (40.0)	1 (20.0)	2 (40.0)

Medication burden was defined as the number of standardized active substances recorded on the index respiratory prescription. Global differences in continuous medication burden across diagnostic groups were assessed using the Kruskal-Wallis test ($H = 86.79$; Benjamini-Hochberg adjusted $p<0.001$). Percentages are calculated within each diagnostic group.

Abbreviations: COPD, chronic obstructive pulmonary disease; ENT, ear, nose, and throat; IQR, interquartile range; SD, standard deviation.

Supplementary Table S3. Multivariable logistic regression results for Model A examining factors associated with at least one moderate or major potential drug–drug interaction at the index respiratory prescription.

Predictor	Adjusted OR (95% CI)	*p*-value
**Demographic variables**		
Age <18 years vs. 18--64 years	0.60 (0.30–1.21)	0.151
Age ≥65 years vs. 18--64 years	0.75 (0.41–1.39)	0.364
Male vs. female	1.52 (0.94–2.46)	0.088
**Diagnostic group**		
Other respiratory conditions vs. asthma	1.73 (0.20–14.62)	0.616
Other chronic or unspecified respiratory or ENT conditions vs. asthma	1.90 (0.73–4.90)	0.186
COPD or chronic obstructive respiratory disease vs. asthma	**2.90 (1.45–5.77)**	**0.003**
Acute bronchitis or lower respiratory tract infections vs. asthma	0.80 (0.31–2.02)	0.631
Influenza or pneumonia vs. asthma	0.29 (0.06–1.50)	0.14
Acute upper respiratory tract infections vs. asthma	1.31 (0.61–2.82)	0.488
Allergic rhinitis or chronic rhinosinusitis vs. asthma	0.54 (0.19–1.51)	0.241
**Medication burden**		
Number of active substances, per additional substance	**2.69 (2.28–3.17)**	**<0.001**

Model A included age group, sex, assigned respiratory diagnostic group, and medication burden expressed as the continuous number of active substances per index prescription. Reference categories were age 18–64 years, female sex, and asthma. $N = 643$.

Abbreviations: OR, odds ratio; CI, confidence interval; COPD, chronic obstructive pulmonary disease; DDI, drug–drug interaction; ENT, ear, nose, and throat.
